# Evaluation of glycemic traits in susceptibility to COVID-19 risk: a Mendelian randomization study

**DOI:** 10.1186/s12916-021-01944-3

**Published:** 2021-03-24

**Authors:** Shiu Lun Au Yeung, Jie V Zhao, C Mary Schooling

**Affiliations:** 1 School of Public Health, LKS Faculty of Medicine, The University of Hong Kong, 1/F, Patrick Manson Building, 7 Sassoon Road, Hong Kong, SAR China; 2 School of Public Health and Health Policy, City University of New York, New York, USA

**Keywords:** COVID-19, Glucose, Glycated hemoglobin, Mendelian randomization, Type 2 diabetes

## Abstract

**Background:**

Observational studies suggest poorer glycemic traits and type 2 diabetes associated with coronavirus disease 2019 (COVID-19) risk although these findings could be confounded by socioeconomic position. We conducted a two-sample Mendelian randomization to clarify their role in COVID-19 risk and specific COVID-19 phenotypes (hospitalized and severe cases).

**Method:**

We identified genetic instruments for fasting glucose (*n* = 133,010), 2 h glucose (*n* = 42,854), glycated hemoglobin (*n* = 123,665), and type 2 diabetes (74,124 cases and 824,006 controls) from genome wide association studies and applied them to COVID-19 Host Genetics Initiative summary statistics (17,965 COVID-19 cases and 1,370,547 population controls). We used inverse variance weighting to obtain the causal estimates of glycemic traits and genetic predisposition to type 2 diabetes in COVID-19 risk. Sensitivity analyses included MR-Egger and weighted median method.

**Results:**

We found genetic predisposition to type 2 diabetes was not associated with any COVID-19 phenotype (OR: 1.00 per unit increase in log odds of having diabetes, 95%CI 0.97 to 1.04 for overall COVID-19; OR: 1.02, 95%CI 0.95 to 1.09 for hospitalized COVID-19; and OR: 1.00, 95%CI 0.93 to 1.08 for severe COVID-19). There were no strong evidence for an association of glycemic traits in COVID-19 phenotypes, apart from a potential inverse association for fasting glucose albeit with wide confidence interval.

**Conclusion:**

We provide some genetic evidence that poorer glycemic traits and predisposition to type 2 diabetes unlikely increase the risk of COVID-19. Although our study did not indicate glycemic traits increase severity of COVID-19, additional studies are needed to verify our findings.

**Supplementary Information:**

The online version contains supplementary material available at 10.1186/s12916-021-01944-3.

## Background

Coronavirus disease 19 (COVID-19) has become a major global health threat. While randomized controlled trials have been quickly conducted to identify possible treatments for COVID-19 [1], other observational studies focus on factors related to increased susceptibility to COVID-19 risk or its severity. Hyperglycemia and type 2 diabetes diagnosis have been associated with increased risk of COVID-19 or with complications in previous observational studies [2–5]. However, it is unclear whether these associations indicate causal targets of intervention due to the possibility of confounding and bias. Furthermore, these observational studies primarily used patient data, and hence, it is unclear whether the observed associations apply to non-hospitalized cases, i.e., less severe and likely asymptomatic cases which may account for the majority of the COVID-19 cases [6].

The use of Mendelian randomization studies helps circumvent the limitations of previous observational studies due to its resistance to confounding by the use of genetic variants randomly allocated at conception to proxy exposures [7]. A recent Mendelian randomization study suggested a role of diabetes in increased ACE2 lung expression, the receptor for SARS-CoV-2 [8], and hence an increased risk of COVID-19. However, an association with a “surrogate endpoint”, i.e., ACE2 expression, does not always indicate a causal effect on the outcome, here COVID-19. To address the causal role of glycemic traits and type 2 diabetes in COVID-19, we conducted a Mendelian randomization study using summary statistics from relevant genome wide association studies (GWAS) of glycemic traits, type 2 diabetes, and COVID-19 [9–13]. We also included subtypes of COVID-19 phenotypes, including hospitalized cases and severe cases, as previous studies also used these more severe COVID-19 phenotypes as outcomes.

## Methods

This is a 2 sample Mendelian randomization study which used summary statistics from relevant GWAS [7]. There are three assumptions. First, the genetic instruments predict the exposure. Second, the genetic instruments are independent of confounders of the exposure-outcome relation; this assumption has been demonstrated empirically in previous studies [14]. Third, the genetic instruments’ effect on the outcome, if any, is only via its relation with the exposure, here glycemic traits and type 2 diabetes.

### Exposure GWAS—glycemic traits and type 2 diabetes

We extracted genetic instruments, i.e., single nucleotide polymorphism (SNP), for glycemic traits from MAGIC GWAS summary statistics, one of the largest genetic consortia on glycemic traits where these GWAS were conducted among people without diabetes. We restricted the sample to people of European descent, and selected instruments strongly (*p* value < 5 × 10^−8^) and independently (not in linkage disequilibrium (LD, *r*^2^ < 0.001 based on European population reference panel) associated with the phenotypes. Specifically, instruments for fasting glucose were obtained from a GWAS of up to 133,010 participants [10]. Instruments for 2 h glucose were obtained from a GWAS of up to 42,854 participants [10]. Instruments for glycated hemoglobin (HbA1c) were obtained from a GWAS of 123,665 participants of European ancestry [13], out of the original GWAS comprised of 159,940 participants of mixed ancestries, as previously [15]. Mean age in these GWAS was around 50 years, with similar proportion of men and women. These GWAS adjusted for age and sex, study-specific covariates, and used genomic control.

Instruments for type 2 diabetes were obtained from the DIAMANTE consortium (*p* value < 5 × 10^− 8^ and not in LD (r^2^ < 0.001)), one of the largest GWAS consortia on type 2 diabetes [12], which comprised 898,130 participants (74,124 cases and 824,006 controls) of European descent [12]. Type 2 diabetes was defined in multiple ways, such as fasting glucose (≥7.0 mmol/L), previous diagnosis of type 2 diabetes, case ascertainment from electronic health records, and use of anti-diabetic medication. The mean age of cases was around 55 years. The GWAS had similar proportions of men and women. The GWAS adjusted for study-specific covariates and controlled for population stratification with genomic control to reduce confounding by ethnicity.

Additional file 1: Table S1 shows the list of genetic instruments used in this study. We calculated the variance of the exposures explained by each instrument using established equations for continuous and binary exposures [16, 17], as shown in Additional file 1: Table S1.

### Outcome GWAS—COVID-19 phenotypes

We extracted summary statistics of all three COVID-19 phenotypes from the most recent data freeze at the time of analyses (Round 4, October 2020) in COVID-19 Host Genetics Initiative (www.covid19hg.org), accessed on November 3, 2020 [11], using rs number or position (Genome Reference Consortium Human Build 37). The COVID-19 Host Genetics Initiative is an initiative comprised of several epidemiologic studies of various designs, such as UK Biobank, deCODE, and FinnGen but we excluded 23andMe study given data from this study were not included in the summary statistics. These phenotypes included any COVID-19 cases, hospitalized COVID-19 cases, and severe COVID cases. In brief, any COVID-19 was defined as having laboratory-confirmed SARS-CoV-2 infection, confirmed COVID-19 from electronic health records/doctor diagnosis, or self-reported COVID-19 positive (17,965 cases and 1,370,547 population controls). Hospitalized COVID was defined as having hospitalized laboratory-confirmed SARS-CoV-2 infection or hospitalization due to corona-related symptom (7885 cases and 961,804 population controls without COVID-19). Very severe respiratory confirmed COVID-19 was defined as having hospitalized laboratory-confirmed SARS-CoV-2 infection, with respiratory support or death (4336 cases and 623,902 population controls without COVID-19). The GWAS was adjusted for sex, age, age^2^, age*sex, principal components and study-specific covariates. Additional file 1: Table S2 shows the instruments’ association with COVID-19 phenotypes. Details of COVID-19 Host Genetics Initiative, such as case ascertainment and contributing studies can be found in Additional file 1: Tables S3–S6.

### Exposures

The exposures were HbA1c (%), fasting glucose (mmol/L), 2 h glucose (mmol/L), and predisposition to type 2 diabetes (per unit increase in log odds of having diabetes).

### Outcomes

The primary outcome was any COVID-19 cases. The secondary outcomes were hospitalized COVID-19 and severe COVID-19 cases.

### Pleiotropic effects

Given a previous Mendelian randomization study suggested a possible role of body mass index (BMI) and smoking on risk of severe COVID-19, we explored the association of genetic instruments of glycemic traits and predisposition to type 2 diabetes in respective genome wide association studies (GIANT consortium for BMI and GSCAN for cigarettes smoked per day) [18, 19], where we considered evidence of pleiotropy if the SNP association reached genome wide significance (*p* value < 5 × 10^−8^).

### Statistical analyses

We approximated the F statistics of each instrument to assess potential weak instrument bias, where higher F statistics indicated lower risk of weak instrument bias [20]. We assessed the association of genetically predicted glycemic traits and predisposition to type 2 diabetes with COVID-19 phenotypes using inverse variance weighting with multiplicative random effects. We also reported heterogeneity of the Wald ratios, i.e., the genetic association with the outcome divided by the genetic association with the exposure, from the Cochrane Q statistics and the MR-Egger intercept *p* value as indicators of potential pleiotropy of the included instruments. We conducted sensitivity analyses using the weighted median and MR-Egger [21, 22].

### Power calculation

We calculated the variance of the exposures explained by each instrument using an established approximation for continuous and binary exposures [16, 17], as shown in Additional file 1: Table S1. We estimated power using the approximation that the sample size for an MR study is the sample size for exposure on outcome divided by the variance of the exposures [23, 24], as shown in Additional file 1: Table S7.

All analyses were performed using R Version 3.6.1 (R Core Team (2019). R: A language and environment for statistical computing. R Foundation for Statistical Computing, Vienna, Austria. https://www.R-project.org/) and the R package (“TwoSampleMR”) [9].

### Ethics approval

This study only used publicly available data and hence no ethics approval was required.

## Results

Up to 34 genetic instruments for HbA1c, 33 instruments for fasting glucose, 7 instruments for 2 h glucose, and 156 instruments for predisposition to type 2 diabetes were used in this study. All instruments had an F statistic > 10, implying weak instrument bias is less likely (Additional file 1: Table S1). Forty-two (42) SNPs were associated with BMI and none of the SNPs were associated with cigarettes smoked per day. Hence, these SNPs were excluded in the sensitivity analyses.

**Fig. 1:**
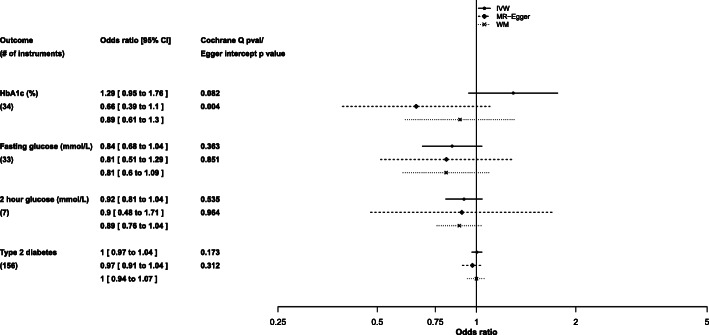
Association of genetically predicted glycemic traits and genetic predisposition to type 2 diabetes on risk of COVID-19 using Mendelian randomization

**Fig. 2:**
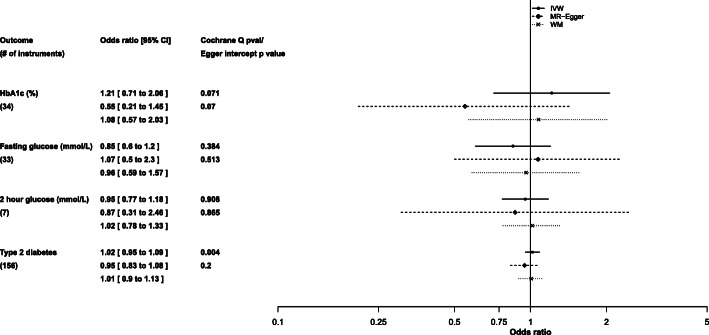
Association of genetically predicted glycemic traits and genetic predisposition to type 2 diabetes on risk of hospitalized COVID-19 using Mendelian randomization

**Fig. 3:**
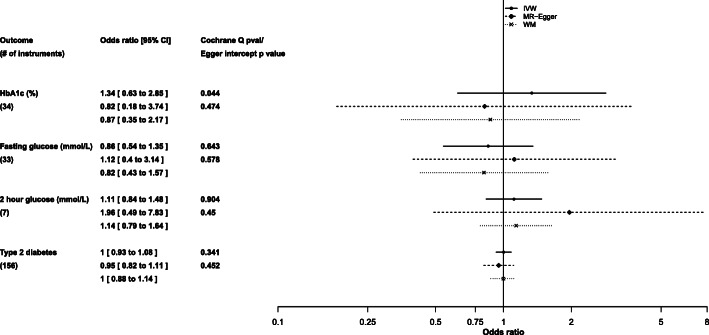
Association of genetically predicted glycemic traits and genetic predisposition to type 2 diabetes on risk of severe COVID-19 using Mendelian randomization

Figure 1 shows the association of genetically predicted glycemic traits and genetic predisposition to type 2 diabetes on risk of COVID-19. There was no strong evidence for an effect of glycemic traits on COVID-19 risk, apart from fasting glucose, where higher fasting glucose appeared to be associated with lower risk of being a COVID-19 case although the estimates had wide confidence intervals. Higher predisposition to type 2 diabetes was not associated with COVID-19 risk, with estimates close to null. The Cochrane Q statistics test and MR-Egger intercept test where there was no strong evidence for heterogeneity, apart from HbA1c where there were signs of horizontal pleiotropy based on the MR-Egger intercept (*p* value: 0.004). However, the corresponding estimates from MR-Egger and weighted median were not always consistent with the main analyses, but with wide confidence intervals. Figures 2 and 3 show the association of genetically predicted glycemic traits and genetic predisposition to type 2 diabetes on risk of hospitalized COVID-19 and severe COVID-19, which indicated no strong evidence for an effect of glycemic traits and predisposition to type 2 diabetes on these more severe COVID-19 phenotypes. There were signs of pleiotropy in some of the analyses, such as for predisposition to type 2 diabetes (Cochrane Q test *p* value: 0.004) related to hospitalized COVID-19 and for HbA1c related to severe COVID-19 (Cochrane *Q* test *p* value: 0.04).

Exclusion of SNPs related to BMI did not change the conclusion regardless of the COVID-19 phenotypes (Additional file 1: Figures S1-S3).

## Discussion

This Mendelian randomization study provides potentially more credible evidence concerning the role of glycemic traits and predisposition to type 2 diabetes in COVID-19 risk and provides no strong evidence for a causal role of hyperglycemia in increasing risk of COVID-19 or its severity. In particular, the estimates for genetic predisposition to type 2 diabetes were close to null. This is consistent with a previous Mendelian randomization study using data from the previous data freeze (Release 3, June 2020) with smaller sample size for hospitalized and severe COVID-19 cases [25]. Our study adds by showing no strong evidence for an association of glycemic traits in these COVID-19 phenotypes using larger case numbers. Furthermore, our study indicated that glycemic traits and predisposition to type 2 diabetes unlikely have a strong role in increasing susceptibility to overall COVID-19, which may be more relevant to the general population given the majority of COVID-19 cases were not severe [26].

Exploring underlying causes of COVID-19 is important for efficient allocation of public health resources to reduce related infections in the population. The recent OpenSAFELY study, using electronic health records of more than 17 million people in the UK, showed that older age, being a male, being obese, lower socio-economic position, and diabetes were associated with higher risk of COVID-19 related death [4]. Although some of these factors may be causal, such as higher body mass index, some may be only risk factors, such as diabetes [25], perhaps because of its association with lower socio-economic position. Furthermore, some findings could be a reflection of bias, such as the paradoxically lower risk of COVID-19 deaths among current smokers, which was not supported by Mendelian randomization which showed a detrimental effect of smoking on COVID-19 [25]. Nevertheless, observational studies provide insights regarding potential causes of COVID-19 but the findings should be further validated using Mendelian randomization when more severe cases have accumulated in the COVID-19 Host Genetics Initiative or using genetic risk scores in the UK Biobank to maximize statistical power [27]. It is also possible that glycemic traits/type 2 diabetes may affect risk of COVID-19 in specific subgroups, perhaps related to underlying genetic susceptibility to COVID-19. However, these dimensions of vulnerability remain to be identified.

One of the motivating reasons for this study is that a previous Mendelian randomization study suggested a possible role of type 2 diabetes in increased risk of COVID-19 [8]. However, that study only used ACE2 expression in the lung as a surrogate outcome, and may not necessarily translate to an effect on the actual outcome, i.e., SARS-CoV-2 infection. This difference has been demonstrated by the debates related to ACE inhibitors where concerns were raised over its use given the possibility of increasing ACE2 expression [28] although subsequent evidence suggests that ACE inhibitors do not appear to increase the risk of COVID-19 [29].

Previous observational studies suggested a possible link of diabetes in infection risk due to possible impairment on the immune system [30, 31], and hence may be relevant to COVID-19 susceptibility and its severity [2–5]. However, the discrepancies between these findings and our study may be indicative of confounding by obesity, or perhaps by medications such as metformin use which were potentially related to lower mortality among those who were admitted to hospital due to COVID-19 [32]. This would warrant further investigations such as using relevant genetic instruments for these medications to assess their impact on COVID-19 severity [33].

Although we used Mendelian randomization which is more resistant to confounding, there are some limitations. Mendelian randomization relies on assumptions for valid inference [7]. We used genetic instruments extracted from published GWAS to reduce risk of weak instrument bias. There was some evidence for pleiotropy in our analyses, indicating the inverse variance weighting analyses may not be valid although the conclusion from MR-Egger remains unchanged. These SNPs do not appear to affect infectious disease risk based on PhenoScanner, a curated database on genetic effects [34], although some SNPs were related to immune markers such as eosinophil and lymphocytes [35] while other SNPs were related to BMI. Nevertheless, pleiotropic effects may have partly explained why estimates from sensitivity analyses were not always directionally consistent for these analyses. Our findings should be verified in future studies when larger number of cases accumulated in the COVID-19 genetic consortium. Mendelian randomization studies are also vulnerable to selection bias and this may partly explain the potential “inverse” association of glucose with COVID phenotypes due to potential competing risk of survival [36]. In this study, we assessed genetic predisposition of type 2 diabetes in COVID-19 phenotypes, and hence, we were not able to assess the impact of type 2 diabetes diagnosis on COVID-19 susceptibility as many of the participants in the COVID-19 GWAS may not have developed type 2 diabetes [37]. As such, the null findings did not directly rule out the possibility of a role of type 2 diabetes in COVID-19 susceptibility. However, the inconsistent findings across glycemic traits and genetic predisposition to type 2 diabetes may suggest hyperglycemia per se did not appear to have a role in susceptibility to COVID-19 risk and its severity. There are also challenges regarding the identification of cases in the course of this COVID-19 pandemic. For example, some of the controls could potentially be “cases” which were not identified given the majority of the cases are asymptomatic or because they were not being tested because of particular social distancing policies targeting high risk groups. This misclassification would also inevitably bias our estimates towards null [6] although this is a challenge also applicable to other COVID-19 studies such as estimation of the case fatality risk [38]. Furthermore, case ascertainment methods were not the same in different cohorts (Additional file 1: Tables S3–S6) but we were unable to explore how these differences may influence our findings because we only had access to overall summary statistics. Lastly, our case number for secondary outcomes was not high and hence we could not rule out the possibility of smaller effects of glycemic traits/ type 2 diabetes on COVID-19 risk. For example, regarding HbA1c, the detectable effect size for per change in standard deviation of HbA1c (~ 0.6% based on our previous study in UK Biobank [33]), would be an odds ratio of 1.15 for any COVID-19 cases, 1.25 for hospitalized COVID-19 cases, and 1.35 for severe COVID-19 cases).

## Conclusions

Our Mendelian randomization study suggested glycemic traits and type 2 diabetes do not appear to increase the risk of COVID-19. As such, our study may imply the observed associations of diabetes with COVID-19 may be at least partly due to people with diabetes being vulnerable to COVID-19 for structural reasons, such as precarious employment or crowded housing [39]. Our study also showed that there is no clear evidence of glycemic traits and type 2 diabetes in increasing the risk of more severe COVID-19. However, these findings should be replicated when larger genetic studies become available.

## Supplementary Information

**Additional file 1: Table S1.** Genetic instruments used in this Mendelian randomization study. **Table S2.** Genetic instruments’ associations with COVID-19 phenotypes. **Table S3.** Studies related to COVID-19 analysis, as per extracted from COVID-19 Host Genetics Initiative (https://www.covid19hg.org/). **Table S4.** Studies related to hospitalized COVID-19 analysis, as per extracted from COVID-19 Host Genetics Initiative (https://www.covid19hg.org/). **Table S5.** Studies related to severe COVID-19 analysis, as per extracted from COVID-19 Host Genetics Initiative (https://www.covid19hg.org/). **Table S6.** Definition of the COVID-19 phenotypes, as per extracted from COVID-19 Host Genetics Initiative (https://www.covid19hg.org/). **Table S7.** Minimum detectable odds ratio per standard deviation of the exposure in this Mendelian randomization, at 80% power and 5% statistical significance. **Figure S1.** Association of genetically predicted glycemic traits and genetic predisposition to type 2 diabetes on risk of COVID-19 using Mendelian randomization, excluding instruments related to body mass index. **Figure S2.** Association of genetically predicted glycemic traits and genetic predisposition to type 2 diabetes on risk of hospitalized COVID-19 using Mendelian randomization excluding instruments related to body mass index. **Figure S3.** Association of genetically predicted glycemic traits and genetic predisposition to type 2 diabetes on risk of severe COVID-19 using Mendelian randomization excluding instruments related to body mass index.

## Data Availability

The data used in this study were publicly available and can be accessed via the links described in the Acknowledgement and the references in the manuscript.
