# Supplementary material for: Evaluation of glycemic traits in susceptibility to COVID-19 risk: a Mendelian randomization study
Source: BMC Med. 2021 Mar 24;19:72. doi: 10.1186/s12916-021-01944-3 (PMC7987511; doi:10.1186/s12916-021-01944-3)
Supplement: Supplementary file 1 — Additional file 1: Table S1. Genetic instruments used in this Mendelian randomization study. Table S2. Genetic instruments’ associations with COVID-19 phenotypes. Table S3. Studies related to COVID-19 analysis, as per extracted from COVID-19 Host Genetics Initiative (https://www.covid19hg.org/). Table S4. Studies related to hospitalized COVID-19 analysis, as per extracted from COVID-19 Host Genetics Initiative (https://www.covid19hg.org/). Table S5. Studies related to severe COVID-19 analysis, as per extracted from COVID-19 Host Genetics Initiative (https://www.covid19hg.org/). Table S6. Definition of the COVID-19 phenotypes, as per extracted from COVID-19 Host Genetics Initiative (https://www.covid19hg.org/). Table S7. Minimum detectable odds ratio per standard deviation of the exposure in this Mendelian randomization, at 80% power and 5% statistical significance. Figure S1. Association of genetically predicted glycemic traits and genetic predisposition to type 2 diabetes on risk of COVID-19 using Mendelian randomization, excluding instruments related to body mass index. Figure S2. Association of genetically predicted glycemic traits and genetic predisposition to type 2 diabetes on risk of hospitalized COVID-19 using Mendelian randomization excluding instruments related to body mass index. Figure S3. Association of genetically predicted glycemic traits and genetic predisposition to type 2 diabetes on risk of severe COVID-19 using Mendelian randomization excluding instruments related to body mass index. [file 12916_2021_1944_MOESM1_ESM.docx]

**Additional file 1: Table S1: Genetic instruments used in this Mendelian randomization study**

| SNP | Effect allele | Other allele | Beta/  log odds | Effect allele frequency | P value | F statistics | Variance explained (r^2^) | Phenotype |
| --- | --- | --- | --- | --- | --- | --- | --- | --- |
| rs1046896 | T | C | 0.028 | 0.3162 | 4.46E-64 | 271.3 | 0.00219 | HbA1c |
| rs10774625 | G | A | 0.009 | 0.5056 | 1.46E-08 | 31.6 | 0.00026 | HbA1c |
| rs10830963 | G | C | 0.02 | 0.2938 | 2.23E-23 | 100.0 | 0.00081 | HbA1c |
| rs11248914 | T | C | 0.014 | 0.647 | 2.56E-14 | 54.3 | 0.00044 | HbA1c |
| rs11558471 | A | G | 0.015 | 0.6745 | 1.38E-19 | 77.9 | 0.00063 | HbA1c |
| rs11603334 | G | A | 0.012 | 0.815 | 6.85E-09 | 32.7 | 0.00026 | HbA1c |
| rs11708067 | A | G | 0.013 | 0.7542 | 1.42E-12 | 46.8 | 0.00038 | HbA1c |
| rs11964178 | A | G | 0.01 | 0.5666 | 6.38E-10 | 39.1 | 0.00032 | HbA1c |
| rs12621844 | T | C | 0.01 | 0.5999 | 1.87E-08 | 30.9 | 0.00025 | HbA1c |
| rs13134327 | A | G | 0.013 | 0.3335 | 2.64E-15 | 58.5 | 0.00047 | HbA1c |
| rs1558902 | A | T | 0.01 | 0.4128 | 3.27E-8 | 27.7 | 0.00022 | HbA1c |
| rs17509001 | C | T | 0.018 | 0.1576 | 1.94E-15 | 61.2 | 0.00050 | HbA1c |
| rs17533903 | A | G | 0.015 | 0.2428 | 5.27E-12 | 46.5 | 0.00038 | HbA1c |
| rs17747324 | C | T | 0.015 | 0.2489 | 6.12E-11 | 42.5 | 0.00034 | HbA1c |
| rs1800562 | G | A | 0.04 | 0.9279 | 4.67E-28 | 123.5 | 0.00100 | HbA1c |
| rs2383208 | A | G | 0.014 | 0.7992 | 7.04E-12 | 44.4 | 0.00036 | HbA1c |
| rs267738 | T | G | 0.011 | 0.7701 | 2.59E-09 | 33.5 | 0.00027 | HbA1c |
| rs282587 | G | A | 0.019 | 0.1513 | 1.70E-12 | 49.5 | 0.00040 | HbA1c |
| rs3782123 | C | A | 0.013 | 0.3205 | 1.51E-10 | 42.3 | 0.00034 | HbA1c |
| rs4607517 | A | G | 0.031 | 0.2017 | 8.76E-38 | 166.8 | 0.00135 | HbA1c |
| rs4737009 | A | G | 0.021 | 0.2531 | 4.48E-27 | 110.3 | 0.00089 | HbA1c |
| rs4745982 | T | G | 0.095 | 0.8726 | 2.87E-65 | 287.8 | 0.00232 | HbA1c |
| rs4820268 | G | A | 0.016 | 0.4606 | 1.40E-22 | 88.6 | 0.00072 | HbA1c |
| rs560887 | C | T | 0.028 | 0.6843 | 1.48E-58 | 242.0 | 0.00195 | HbA1c |
| rs579459 | C | T | 0.011 | 0.2389 | 9.42E-09 | 33.5 | 0.00027 | HbA1c |
| rs592423 | A | C | 0.009 | 0.4566 | 3.96E-08 | 28.0 | 0.00023 | HbA1c |
| rs6474359 | T | C | 0.044 | 0.953 | 1.50E-16 | 68.9 | 0.00056 | HbA1c |
| rs7040409 | C | G | 0.028 | 0.8953 | 2.56E-14 | 57.3 | 0.00046 | HbA1c |
| rs7616006 | A | G | 0.01 | 0.5744 | 5.07E-10 | 34.6 | 0.00028 | HbA1c |
| rs8192675 | T | C | 0.011 | 0.6906 | 1.38E-11 | 41.9 | 0.00034 | HbA1c |
| rs837763 | T | C | 0.017 | 0.5548 | 1.68E-28 | 112.9 | 0.00091 | HbA1c |
| rs857691 | T | C | 0.019 | 0.2715 | 3.97E-25 | 100.0 | 0.00081 | HbA1c |
| rs9818758 | A | G | 0.012 | 0.2028 | 7.74E-10 | 36.0 | 0.00029 | HbA1c |
| rs9914988 | A | G | 0.013 | 0.7877 | 2.77E-11 | 42.3 | 0.00034 | HbA1c |
| rs10747083 | A | G | 0.013 | Not reported | 7.92E-09 | 31.9 | 0.00024 | Glucose |
| rs10758593 | G | A | -0.016 | Not reported | 1.76E-13 | 52.9 | 0.00040 | Glucose |
| rs10965250 | G | A | 0.024 | Not reported | 5.11E-18 | 73.5 | 0.00055 | Glucose |
| rs11195502 | C | T | 0.032 | Not reported | 2.61E-18 | 74.8 | 0.00056 | Glucose |
| rs11603334 | G | A | 0.019 | Not reported | 5.78E-12 | 46.0 | 0.00035 | Glucose |
| rs11607883 | G | A | 0.021 | Not reported | 7.62E-24 | 100.0 | 0.00075 | Glucose |
| rs11619319 | A | G | -0.02 | Not reported | 3.93E-17 | 69.4 | 0.00052 | Glucose |
| rs11672660 | C | T | 0.016 | Not reported | 5.51E-09 | 32.7 | 0.00025 | Glucose |
| rs11708067 | A | G | 0.023 | Not reported | 4.53E-19 | 78.3 | 0.00059 | Glucose |
| rs11715915 | C | T | 0.012 | Not reported | 2.45E-08 | 29.8 | 0.00022 | Glucose |
| rs1260326 | C | T | 0.029 | Not reported | 1.12E-43 | 190.7 | 0.00143 | Glucose |
| rs1280 | T | C | 0.026 | Not reported | 2.49E-17 | 70.3 | 0.00053 | Glucose |
| rs13266634 | C | T | 0.029 | Not reported | 9.45E-37 | 159.0 | 0.00119 | Glucose |
| rs16913693 | T | G | 0.043 | Not reported | 3.63E-11 | 42.4 | 0.00032 | Glucose |
| rs17168486 | C | T | -0.031 | Not reported | 8.63E-29 | 122.6 | 0.00092 | Glucose |
| rs174576 | C | A | 0.02 | Not reported | 4.91E-20 | 82.6 | 0.00062 | Glucose |
| rs2108349 | A | G | -0.015 | Not reported | 4.61E-12 | 46.5 | 0.00035 | Glucose |
| rs2191349 | G | T | -0.029 | Not reported | 1.12E-43 | 190.7 | 0.00143 | Glucose |
| rs3783347 | G | T | 0.017 | Not reported | 3.11E-11 | 42.8 | 0.00032 | Glucose |
| rs3829109 | G | A | 0.017 | Not reported | 1.52E-10 | 39.6 | 0.00030 | Glucose |
| rs4502156 | T | C | 0.022 | Not reported | 5.56E-26 | 109.8 | 0.00082 | Glucose |
| rs479661 | A | G | -0.019 | Not reported | 5.78E-12 | 46.0 | 0.00035 | Glucose |
| rs4869272 | C | T | -0.018 | Not reported | 1.40E-16 | 66.9 | 0.00050 | Glucose |
| rs560887 | C | T | 0.071 | Not reported | 1.01E-177 | 806.6 | 0.00603 | Glucose |
| rs6072275 | G | A | -0.016 | Not reported | 5.51E-09 | 32.7 | 0.00025 | Glucose |
| rs6113722 | G | A | 0.035 | Not reported | 2.00E-11 | 43.6 | 0.00033 | Glucose |
| rs6975024 | T | C | -0.061 | Not reported | 1.59E-98 | 442.4 | 0.00332 | Glucose |
| rs749067 | C | T | -0.017 | Not reported | 5.49E-15 | 59.7 | 0.00045 | Glucose |
| rs7633675 | T | G | -0.013 | Not reported | 7.92E-09 | 31.9 | 0.00024 | Glucose |
| rs7756992 | G | A | 0.014 | Not reported | 5.75E-10 | 37.1 | 0.00028 | Glucose |
| rs7903146 | C | T | -0.022 | Not reported | 2.44E-20 | 84.0 | 0.00063 | Glucose |
| rs882020 | C | T | -0.021 | Not reported | 1.28E-12 | 49.0 | 0.00037 | Glucose |
| rs983309 | G | T | -0.026 | Not reported | 1.65E-15 | 62.1 | 0.00047 | Glucose |
| rs1019503 | A | G | 0.063 | Not reported | 5.10E-09 | 32.8 | 0.00076 | 2h glucose |
| rs10934647 | T | C | -0.085 | Not reported | 3.11E-11 | 42.8 | 0.00100 | 2h glucose |
| rs11672660 | T | C | 0.12 | Not reported | 5.11E-18 | 73.5 | 0.00171 | 2h glucose |
| rs11782386 | T | C | -0.099 | Not reported | 2.88E-09 | 33.9 | 0.00079 | 2h glucose |
| rs12255372 | T | G | 0.092 | Not reported | 7.37E-13 | 50.1 | 0.00117 | 2h glucose |
| rs6547829 | T | C | 0.11 | Not reported | 3.53E-09 | 33.5 | 0.00078 | 2h glucose |
| rs6975024 | T | C | -0.1 | Not reported | 2.05E-10 | 39.1 | 0.00091 | 2h glucose |
| rs1005752 | A | C | 0.077 | 0.715 | 2.50E-29 | 119.0 | 0.00037 | T2D |
| rs10096633 | C | T | 0.068 | 0.877 | 1.10E-12 | 50.3 | 0.00015 | T2D |
| rs10097617 | T | C | 0.039 | 0.485 | 3.30E-11 | 28.7 | 0.00012 | T2D |
| rs10193538 | T | G | 0.039 | 0.61 | 8.90E-09 | 28.1 | 0.00011 | T2D |
| rs10195252 | T | C | 0.068 | 0.586 | 6.00E-25 | 201.3 | 0.00034 | T2D |
| rs10228066 | T | C | 0.068 | 0.537 | 1.10E-28 | 90.3 | 0.00035 | T2D |
| rs10406431 | A | G | 0.049 | 0.563 | 9.60E-14 | 100.8 | 0.00018 | T2D |
| rs1061810 | A | C | 0.049 | 0.288 | 6.00E-13 | 45.2 | 0.00015 | T2D |
| rs10750397 | A | G | 0.049 | 0.282 | 8.30E-13 | 45.2 | 0.00015 | T2D |
| rs10811660 | G | A | 0.239 | 0.828 | 1.40E-115 | 561.8 | 0.00246 | T2D |
| rs10830963 | G | C | 0.095 | 0.277 | 4.80E-43 | 189.4 | 0.00055 | T2D |
| rs10842994 | C | T | 0.077 | 0.805 | 4.10E-20 | 116.9 | 0.00028 | T2D |
| rs10882101 | T | C | 0.058 | 0.587 | 1.40E-08 | 36.6 | 0.00025 | T2D |
| rs10938398 | A | G | 0.049 | 0.429 | 3.60E-12 | 44.4 | 0.00018 | T2D |
| rs10954772 | T | C | 0.039 | 0.314 | 1.80E-09 | 28.7 | 0.00010 | T2D |
| rs10974438 | C | A | 0.049 | 0.357 | 1.50E-14 | 45.2 | 0.00017 | T2D |
| rs11063028 | C | T | 0.058 | 0.18 | 8.50E-11 | 64.5 | 0.00015 | T2D |
| rs11257655 | T | C | 0.086 | 0.218 | 1.50E-32 | 152.0 | 0.00039 | T2D |
| rs1127215 | C | T | 0.049 | 0.584 | 1.60E-13 | 100.8 | 0.00018 | T2D |
| rs11496066 | T | C | 0.077 | 0.818 | 1.10E-08 | 29.5 | 0.00027 | T2D |
| rs115505614 | T | C | 0.174 | 0.0499 | 1.30E-30 | 133.2 | 0.00044 | T2D |
| rs11642430 | G | C | 0.039 | 0.399 | 2.20E-09 | 63.9 | 0.00011 | T2D |
| rs11680058 | A | G | 0.058 | 0.863 | 1.40E-08 | 36.6 | 0.00012 | T2D |
| rs11688682 | G | C | 0.049 | 0.728 | 4.20E-09 | 44.4 | 0.00014 | T2D |
| rs11699802 | C | T | 0.039 | 0.536 | 1.80E-11 | 28.7 | 0.00012 | T2D |
| rs11708067 | A | G | 0.086 | 0.772 | 5.20E-32 | 152.0 | 0.00040 | T2D |
| rs11709077 | G | A | 0.131 | 0.877 | 1.80E-36 | 135.9 | 0.00056 | T2D |
| rs11759026 | G | A | 0.068 | 0.232 | 2.40E-18 | 88.6 | 0.00025 | T2D |
| rs11842871 | G | T | 0.039 | 0.735 | 1.20E-08 | 28.7 | 0.00009 | T2D |
| rs12001437 | C | T | 0.039 | 0.372 | 2.80E-10 | 28.7 | 0.00011 | T2D |
| rs12140153 | G | T | 0.068 | 0.905 | 1.30E-08 | 31.9 | 0.00012 | T2D |
| rs1260326 | C | T | 0.068 | 0.607 | 6.50E-25 | 201.3 | 0.00033 | T2D |
| rs12640250 | C | A | 0.039 | 0.715 | 3.70E-08 | 63.9 | 0.00010 | T2D |
| rs12719778 | T | C | 0.039 | 0.538 | 5.00E-09 | 63.9 | 0.00012 | T2D |
| rs12811407 | A | G | 0.049 | 0.331 | 1.70E-12 | 45.2 | 0.00016 | T2D |
| rs12920022 | A | T | 0.049 | 0.158 | 3.40E-09 | 45.2 | 0.00010 | T2D |
| rs1296328 | A | C | 0.039 | 0.446 | 3.50E-08 | 28.1 | 0.00012 | T2D |
| rs13041756 | C | T | 0.058 | 0.107 | 1.40E-08 | 36.6 | 0.00010 | T2D |
| rs13262861 | C | A | 0.068 | 0.829 | 4.00E-12 | 50.3 | 0.00020 | T2D |
| rs13426680 | A | G | 0.086 | 0.937 | 6.70E-10 | 53.7 | 0.00013 | T2D |
| rs1359790 | G | A | 0.086 | 0.72 | 2.40E-31 | 149.3 | 0.00045 | T2D |
| rs1377807 | C | G | 0.049 | 0.312 | 4.20E-13 | 45.2 | 0.00016 | T2D |
| rs1412234 | C | T | 0.039 | 0.323 | 1.90E-10 | 28.7 | 0.00010 | T2D |
| rs141521721 | A | C | 0.122 | 0.0236 | 2.70E-08 | 35.8 | 0.00010 | T2D |
| rs1421085 | C | T | 0.122 | 0.415 | 3.10E-84 | 328.5 | 0.00110 | T2D |
| rs1426371 | G | A | 0.049 | 0.739 | 8.20E-12 | 45.2 | 0.00014 | T2D |
| rs145678014 | G | T | 0.104 | 0.957 | 2.00E-10 | 41.7 | 0.00014 | T2D |
| rs145904381 | T | C | 0.174 | 0.987 | 2.60E-08 | 33.5 | 0.00012 | T2D |
| rs1493694 | T | C | 0.086 | 0.109 | 2.70E-16 | 84.7 | 0.00022 | T2D |
| rs1531583 | T | G | 0.122 | 0.0458 | 3.50E-14 | 59.2 | 0.00020 | T2D |
| rs1561927 | C | T | 0.039 | 0.269 | 1.50E-09 | 28.7 | 0.00009 | T2D |
| rs1562396 | G | A | 0.058 | 0.319 | 9.90E-18 | 65.7 | 0.00022 | T2D |
| rs1580278 | C | A | 0.039 | 0.473 | 2.20E-10 | 63.9 | 0.00012 | T2D |
| rs1708302 | C | T | 0.095 | 0.512 | 1.10E-48 | 185.9 | 0.00069 | T2D |
| rs17122772 | G | C | 0.039 | 0.228 | 1.60E-08 | 28.7 | 0.00008 | T2D |
| rs17168486 | T | C | 0.068 | 0.181 | 2.30E-17 | 90.3 | 0.00021 | T2D |
| rs17250977 | G | A | 0.113 | 0.0376 | 2.00E-11 | 50.9 | 0.00014 | T2D |
| rs17522122 | T | G | 0.039 | 0.474 | 3.20E-09 | 63.9 | 0.00012 | T2D |
| rs17684074 | G | C | 0.039 | 0.74 | 2.90E-08 | 28.7 | 0.00009 | T2D |
| rs17772814 | G | A | 0.077 | 0.915 | 5.40E-10 | 29.5 | 0.00014 | T2D |
| rs17791513 | A | G | 0.095 | 0.932 | 3.10E-14 | 68.2 | 0.00017 | T2D |
| rs1783541 | T | C | 0.058 | 0.204 | 2.00E-14 | 65.7 | 0.00017 | T2D |
| rs17836088 | C | G | 0.058 | 0.217 | 6.70E-14 | 36.6 | 0.00018 | T2D |
| rs1800961 | T | C | 0.166 | 0.0353 | 2.30E-22 | 93.1 | 0.00028 | T2D |
| rs184509201 | C | G | 0.191 | 0.982 | 1.20E-13 | 56.7 | 0.00019 | T2D |
| rs2102278 | G | A | 0.039 | 0.319 | 3.70E-08 | 28.1 | 0.00010 | T2D |
| rs2197973 | T | C | 0.039 | 0.538 | 3.60E-08 | 28.1 | 0.00012 | T2D |
| rs2237895 | C | A | 0.113 | 0.426 | 6.00E-52 | 277.5 | 0.00095 | T2D |
| rs2258238 | T | A | 0.095 | 0.104 | 4.50E-21 | 68.2 | 0.00026 | T2D |
| rs2268078 | A | G | 0.039 | 0.657 | 2.30E-10 | 28.7 | 0.00011 | T2D |
| rs2272163 | C | A | 0.039 | 0.618 | 9.60E-09 | 28.1 | 0.00011 | T2D |
| rs2283220 | A | G | 0.049 | 0.69 | 1.40E-09 | 44.4 | 0.00015 | T2D |
| rs2307111 | T | C | 0.049 | 0.605 | 2.10E-16 | 45.2 | 0.00017 | T2D |
| rs243024 | A | G | 0.058 | 0.46 | 2.50E-20 | 146.5 | 0.00026 | T2D |
| rs2767036 | C | A | 0.039 | 0.291 | 3.30E-08 | 28.1 | 0.00010 | T2D |
| rs2796441 | G | A | 0.068 | 0.592 | 4.40E-24 | 88.6 | 0.00034 | T2D |
| rs28505901 | G | A | 0.086 | 0.752 | 6.70E-26 | 84.7 | 0.00042 | T2D |
| rs2972144 | G | A | 0.095 | 0.639 | 2.10E-46 | 185.9 | 0.00064 | T2D |
| rs3111316 | A | G | 0.049 | 0.589 | 6.30E-13 | 44.4 | 0.00018 | T2D |
| rs329122 | A | G | 0.039 | 0.429 | 3.60E-09 | 63.9 | 0.00011 | T2D |
| rs340874 | C | T | 0.068 | 0.556 | 1.60E-22 | 88.6 | 0.00034 | T2D |
| rs34584161 | A | G | 0.049 | 0.76 | 2.20E-10 | 44.4 | 0.00013 | T2D |
| rs34715063 | C | T | 0.095 | 0.124 | 2.30E-19 | 66.9 | 0.00030 | T2D |
| rs348330 | G | A | 0.049 | 0.361 | 2.70E-14 | 45.2 | 0.00017 | T2D |
| rs34965774 | A | G | 0.058 | 0.144 | 2.00E-09 | 36.6 | 0.00013 | T2D |
| rs35352848 | T | C | 0.068 | 0.788 | 1.30E-17 | 50.3 | 0.00023 | T2D |
| rs35895680 | C | A | 0.058 | 0.678 | 2.50E-15 | 64.5 | 0.00023 | T2D |
| rs35999103 | T | C | 0.049 | 0.155 | 9.70E-09 | 25.2 | 0.00009 | T2D |
| rs3751837 | T | C | 0.039 | 0.22 | 1.40E-08 | 28.7 | 0.00008 | T2D |
| rs3768321 | T | G | 0.086 | 0.2 | 2.60E-26 | 149.3 | 0.00036 | T2D |
| rs3798519 | C | A | 0.058 | 0.184 | 2.60E-12 | 36.6 | 0.00016 | T2D |
| rs3802177 | G | A | 0.104 | 0.685 | 1.10E-55 | 231.2 | 0.00071 | T2D |
| rs3845281 | G | A | 0.077 | 0.904 | 2.30E-11 | 66.3 | 0.00016 | T2D |
| rs3887925 | T | C | 0.068 | 0.547 | 3.10E-22 | 88.6 | 0.00034 | T2D |
| rs4238013 | C | T | 0.058 | 0.209 | 3.20E-11 | 64.5 | 0.00017 | T2D |
| rs429358 | T | C | 0.077 | 0.846 | 2.60E-18 | 66.3 | 0.00023 | T2D |
| rs4457053 | G | A | 0.058 | 0.304 | 8.40E-18 | 65.7 | 0.00022 | T2D |
| rs465002 | T | C | 0.104 | 0.742 | 6.10E-38 | 227.0 | 0.00063 | T2D |
| rs4688760 | T | C | 0.039 | 0.684 | 3.50E-10 | 28.7 | 0.00010 | T2D |
| rs4709746 | C | T | 0.058 | 0.868 | 5.80E-09 | 36.6 | 0.00012 | T2D |
| rs474513 | A | G | 0.039 | 0.517 | 8.10E-10 | 63.9 | 0.00012 | T2D |
| rs4804833 | A | G | 0.049 | 0.39 | 7.70E-13 | 44.4 | 0.00017 | T2D |
| rs4925109 | A | G | 0.049 | 0.316 | 2.80E-12 | 44.4 | 0.00016 | T2D |
| rs4929965 | A | G | 0.068 | 0.383 | 4.00E-26 | 90.3 | 0.00033 | T2D |
| rs4932265 | T | C | 0.068 | 0.267 | 4.20E-20 | 88.6 | 0.00027 | T2D |
| rs4946812 | G | A | 0.039 | 0.674 | 8.20E-09 | 63.9 | 0.00010 | T2D |
| rs4977213 | C | T | 0.049 | 0.375 | 9.10E-14 | 45.2 | 0.00017 | T2D |
| rs539515 | C | A | 0.049 | 0.198 | 1.60E-10 | 45.2 | 0.00011 | T2D |
| rs55653563 | A | C | 0.039 | 0.732 | 2.20E-09 | 28.7 | 0.00009 | T2D |
| rs56337234 | C | T | 0.058 | 0.503 | 8.60E-18 | 64.5 | 0.00026 | T2D |
| rs56348580 | G | C | 0.049 | 0.689 | 2.30E-13 | 45.2 | 0.00015 | T2D |
| rs58432198 | C | T | 0.068 | 0.881 | 2.10E-10 | 50.3 | 0.00015 | T2D |
| rs58730668 | T | C | 0.068 | 0.858 | 1.30E-13 | 50.3 | 0.00017 | T2D |
| rs601945 | G | A | 0.058 | 0.178 | 4.70E-08 | 36.6 | 0.00015 | T2D |
| rs60276348 | T | C | 0.049 | 0.14 | 2.60E-08 | 25.2 | 0.00009 | T2D |
| rs61676547 | C | G | 0.058 | 0.192 | 2.90E-11 | 64.5 | 0.00016 | T2D |
| rs62007683 | G | T | 0.039 | 0.653 | 3.10E-08 | 28.1 | 0.00011 | T2D |
| rs62080313 | C | T | 0.058 | 0.123 | 1.00E-08 | 36.6 | 0.00011 | T2D |
| rs62107261 | T | C | 0.113 | 0.954 | 3.80E-12 | 50.0 | 0.00017 | T2D |
| rs62271373 | A | T | 0.086 | 0.0553 | 1.00E-09 | 37.6 | 0.00012 | T2D |
| rs6458354 | C | T | 0.049 | 0.289 | 2.10E-12 | 45.2 | 0.00015 | T2D |
| rs6459733 | G | C | 0.058 | 0.673 | 2.40E-17 | 146.5 | 0.00023 | T2D |
| rs6518681 | G | A | 0.086 | 0.914 | 1.10E-12 | 53.7 | 0.00018 | T2D |
| rs6600191 | T | C | 0.058 | 0.825 | 9.30E-13 | 65.7 | 0.00015 | T2D |
| rs67232546 | T | C | 0.058 | 0.207 | 1.30E-11 | 64.5 | 0.00017 | T2D |
| rs6780171 | A | T | 0.131 | 0.314 | 9.00E-56 | 214.2 | 0.00112 | T2D |
| rs6821438 | A | G | 0.039 | 0.534 | 4.00E-11 | 28.7 | 0.00012 | T2D |
| rs6976111 | A | C | 0.039 | 0.313 | 1.20E-08 | 28.7 | 0.00010 | T2D |
| rs7022807 | G | A | 0.039 | 0.401 | 2.70E-10 | 63.9 | 0.00011 | T2D |
| rs702634 | A | G | 0.049 | 0.69 | 7.70E-14 | 45.2 | 0.00015 | T2D |
| rs71372253 | C | T | 0.077 | 0.0642 | 4.40E-08 | 42.1 | 0.00011 | T2D |
| rs7178762 | C | T | 0.039 | 0.46 | 5.40E-10 | 63.9 | 0.00012 | T2D |
| rs7222481 | C | G | 0.039 | 0.324 | 1.40E-08 | 63.9 | 0.00010 | T2D |
| rs7240767 | C | T | 0.039 | 0.376 | 1.60E-08 | 28.1 | 0.00011 | T2D |
| rs72802342 | C | A | 0.157 | 0.923 | 4.00E-32 | 144.0 | 0.00053 | T2D |
| rs72926932 | C | A | 0.086 | 0.0839 | 1.00E-14 | 54.7 | 0.00017 | T2D |
| rs738408 | T | C | 0.049 | 0.226 | 1.40E-10 | 25.2 | 0.00013 | T2D |
| rs7629630 | A | T | 0.049 | 0.857 | 2.50E-08 | 25.2 | 0.00009 | T2D |
| rs7669833 | T | A | 0.058 | 0.705 | 1.20E-14 | 64.5 | 0.00021 | T2D |
| rs76895963 | T | G | 0.482 | 0.98 | 1.40E-69 | 326.2 | 0.00138 | T2D |
| rs7719891 | G | A | 0.039 | 0.259 | 2.40E-08 | 28.7 | 0.00009 | T2D |
| rs77464186 | A | C | 0.104 | 0.836 | 4.70E-33 | 128.8 | 0.00045 | T2D |
| rs7756992 | G | A | 0.140 | 0.274 | 2.40E-88 | 248.0 | 0.00118 | T2D |
| rs77864822 | A | G | 0.077 | 0.932 | 1.10E-08 | 29.5 | 0.00011 | T2D |
| rs7903146 | T | C | 0.315 | 0.295 | 1.00E-200 | 1786.2 | 0.00624 | T2D |
| rs7987740 | T | C | 0.039 | 0.609 | 4.00E-08 | 28.1 | 0.00011 | T2D |
| rs8010382 | G | A | 0.039 | 0.421 | 6.50E-09 | 63.9 | 0.00011 | T2D |
| rs80147536 | A | T | 0.122 | 0.904 | 2.70E-29 | 118.2 | 0.00039 | T2D |
| rs8107974 | T | A | 0.095 | 0.0769 | 3.30E-15 | 66.9 | 0.00020 | T2D |
| rs878521 | A | G | 0.058 | 0.245 | 1.90E-13 | 64.5 | 0.00019 | T2D |
| rs9379084 | G | A | 0.104 | 0.887 | 3.30E-21 | 81.7 | 0.00033 | T2D |
| rs9494624 | A | G | 0.039 | 0.29 | 6.10E-09 | 28.7 | 0.00010 | T2D |
| rs9563615 | A | T | 0.049 | 0.71 | 6.40E-11 | 44.4 | 0.00015 | T2D |
| rs9860730 | A | G | 0.058 | 0.704 | 4.90E-15 | 64.5 | 0.00021 | T2D |
| rs9873618 | G | A | 0.068 | 0.71 | 4.80E-21 | 88.6 | 0.00029 | T2D |
| rs9957145 | G | A | 0.049 | 0.829 | 8.10E-09 | 25.2 | 0.00010 | T2D |

SNP: Single nucleotide polymorphism; T2D: Type 2 diabetes

**Additional file 1: Table S2: Genetic instruments’ associations with COVID-19 phenotypes**

|  |  |  |  | Any COVID-19 | | | Hospitalized COVID-19 | | Severe COVID-19 | |
| --- | --- | --- | --- | --- | --- | --- | --- | --- | --- | --- |
| SNP | Phenotype | Effect allele | Other allele | EAF | Log odds | SE | Log odds | SE | Log odds | SE |
| rs1046896 | HbA1c | T | C | 0.302 | -0.003 | 0.013 | 0.007 | 0.022 | -0.011 | 0.029 |
| rs10774625 | HbA1c | G | A | 0.564 | 0.010 | 0.014 | 0.013 | 0.025 | 0.020 | 0.037 |
| rs10830963 | HbA1c | G | C | 0.291 | 0.019 | 0.014 | 0.019 | 0.024 | 0.005 | 0.031 |
| rs11248914 | HbA1c | T | C | 0.675 | 0.008 | 0.015 | -0.009 | 0.027 | -0.035 | 0.037 |
| rs11558471 | HbA1c | A | G | 0.669 | 0.009 | 0.014 | -0.035 | 0.023 | -0.044 | 0.030 |
| rs11603334 | HbA1c | G | A | 0.822 | -0.008 | 0.018 | -0.038 | 0.030 | -0.096 | 0.040 |
| rs11708067 | HbA1c | A | G | 0.787 | 0.015 | 0.016 | 0.049 | 0.025 | 0.047 | 0.034 |
| rs11964178 | HbA1c | A | G | 0.545 | 0.006 | 0.013 | 0.017 | 0.021 | 0.015 | 0.028 |
| rs12621844 | HbA1c | T | C | 0.602 | 0.015 | 0.013 | 0.016 | 0.021 | 0.021 | 0.028 |
| rs13134327 | HbA1c | A | G | 0.319 | 0.009 | 0.013 | 0.025 | 0.022 | 0.008 | 0.029 |
| rs1558902 | HbA1c | A | T | 0.407 | 0.012 | 0.014 | 0.015 | 0.021 | -0.020 | 0.028 |
| rs17509001 | HbA1c | C | T | 0.135 | -0.008 | 0.018 | 0.005 | 0.029 | -0.029 | 0.039 |
| rs17533903 | HbA1c | A | G | 0.237 | -0.015 | 0.016 | -0.012 | 0.026 | -0.018 | 0.035 |
| rs17747324 | HbA1c | C | T | 0.218 | 0.022 | 0.017 | 0.037 | 0.028 | 0.100 | 0.041 |
| rs1800562 | HbA1c | G | A | 0.936 | -0.004 | 0.031 | -0.022 | 0.049 | 0.012 | 0.063 |
| rs2383208 | HbA1c | A | G | 0.822 | -0.004 | 0.016 | -0.022 | 0.026 | -0.022 | 0.035 |
| rs267738 | HbA1c | T | G | 0.791 | -0.027 | 0.016 | -0.028 | 0.026 | -0.015 | 0.035 |
| rs282587 | HbA1c | G | A | 0.136 | 0.029 | 0.019 | -0.017 | 0.033 | 0.015 | 0.053 |
| rs3782123 | HbA1c | C | A | 0.281 | -0.004 | 0.015 | -0.005 | 0.025 | -0.006 | 0.031 |
| rs4607517 | HbA1c | A | G | 0.153 | -0.013 | 0.018 | 0.006 | 0.027 | 0.016 | 0.035 |
| rs4737009 | HbA1c | A | G | 0.248 | 0.015 | 0.014 | 0.004 | 0.023 | 0.037 | 0.031 |
| rs4745982 | HbA1c | T | G | 0.914 | -0.026 | 0.026 | -0.057 | 0.047 | -0.018 | 0.071 |
| rs4820268 | HbA1c | G | A | 0.453 | 0.028 | 0.013 | 0.004 | 0.021 | -0.010 | 0.028 |
| rs560887 | HbA1c | C | T | 0.709 | -0.016 | 0.016 | -0.007 | 0.027 | -0.029 | 0.041 |
| rs579459 | HbA1c | C | T | 0.203 | 0.064 | 0.016 | 0.119 | 0.025 | 0.139 | 0.033 |
| rs592423 | HbA1c | A | C | 0.462 | 0.001 | 0.013 | -0.016 | 0.021 | -0.010 | 0.028 |
| rs6474359 | HbA1c | T | C | 0.956 | 0.021 | 0.028 | -0.001 | 0.047 | 0.101 | 0.075 |
| rs7040409 | HbA1c | C | G | 0.926 | 0.021 | 0.024 | 0.052 | 0.039 | 0.021 | 0.054 |
| rs7616006 | HbA1c | A | G | 0.603 | 0.014 | 0.013 | 0.009 | 0.021 | 0.049 | 0.027 |
| rs8192675 | HbA1c | T | C | 0.711 | 0.001 | 0.014 | -0.001 | 0.022 | -0.004 | 0.030 |
| rs837763 | HbA1c | T | C | 0.555 | 0.014 | 0.013 | 0.011 | 0.024 | -0.011 | 0.036 |
| rs857691 | HbA1c | T | C | 0.277 | 0.014 | 0.017 | 0.036 | 0.028 | 0.055 | 0.040 |
| rs9818758 | HbA1c | A | G | 0.184 | 0.024 | 0.017 | 0.030 | 0.027 | 0.047 | 0.036 |
| rs9914988 | HbA1c | A | G | 0.787 | 0.020 | 0.015 | 0.008 | 0.025 | -0.007 | 0.034 |
| rs10747083 | Glucose | A | G | 0.674 | 0.018 | 0.014 | -0.009 | 0.026 | 0.030 | 0.039 |
| rs10758593 | Glucose | G | A | 0.582 | -0.001 | 0.013 | -0.011 | 0.021 | -0.054 | 0.027 |
| rs10965250 | Glucose | G | A | 0.831 | -0.006 | 0.017 | -0.029 | 0.027 | -0.033 | 0.036 |
| rs11195502 | Glucose | C | T | 0.898 | 0.017 | 0.019 | 0.043 | 0.032 | 0.057 | 0.045 |
| rs11603334 | Glucose | G | A | 0.822 | -0.008 | 0.018 | -0.038 | 0.030 | -0.096 | 0.040 |
| rs11607883 | Glucose | G | A | 0.502 | -0.017 | 0.013 | -0.002 | 0.021 | 0.005 | 0.028 |
| rs11619319 | Glucose | A | G | 0.770 | -0.003 | 0.015 | 0.048 | 0.024 | 0.026 | 0.033 |
| rs11672660 | Glucose | C | T | 0.782 | 0.010 | 0.016 | 0.005 | 0.026 | -0.028 | 0.035 |
| rs11708067 | Glucose | A | G | 0.787 | 0.015 | 0.016 | 0.049 | 0.025 | 0.047 | 0.034 |
| rs11715915 | Glucose | C | T | 0.669 | -0.007 | 0.014 | -0.013 | 0.022 | -0.020 | 0.030 |
| rs1260326 | Glucose | C | T | 0.630 | 0.016 | 0.013 | 0.033 | 0.021 | 0.030 | 0.028 |
| rs1280 | Glucose | T | C | 0.868 | -0.017 | 0.017 | -0.017 | 0.029 | -0.021 | 0.039 |
| rs13266634 | Glucose | C | T | 0.674 | 0.008 | 0.014 | -0.034 | 0.023 | -0.032 | 0.030 |
| rs16913693 | Glucose | T | G | 0.962 | -0.076 | 0.031 | -0.100 | 0.052 | -0.076 | 0.084 |
| rs17168486 | Glucose | C | T | 0.804 | -0.020 | 0.017 | -0.012 | 0.028 | -0.027 | 0.036 |
| rs174576 | Glucose | C | A | 0.634 | -0.002 | 0.013 | -0.022 | 0.022 | -0.022 | 0.029 |
| rs2108349 | Glucose | A | G | 0.641 | -0.016 | 0.014 | -0.010 | 0.025 | 0.027 | 0.040 |
| rs2191349 | Glucose | G | T | 0.475 | 0.014 | 0.013 | -0.002 | 0.021 | -0.008 | 0.028 |
| rs3783347 | Glucose | G | T | 0.789 | -0.014 | 0.016 | -0.008 | 0.026 | -0.015 | 0.034 |
| rs3829109 | Glucose | G | A | 0.714 | -0.024 | 0.016 | -0.033 | 0.028 | -0.051 | 0.043 |
| rs4502156 | Glucose | T | C | 0.517 | -0.008 | 0.013 | -0.013 | 0.021 | -0.035 | 0.027 |
| rs479661 | Glucose | A | G | 0.840 | 0.048 | 0.019 | 0.063 | 0.033 | 0.042 | 0.047 |
| rs4869272 | Glucose | C | T | 0.317 | -0.004 | 0.014 | -0.001 | 0.022 | -0.003 | 0.029 |
| rs560887 | Glucose | C | T | 0.709 | -0.016 | 0.016 | -0.007 | 0.027 | -0.029 | 0.041 |
| rs6072275 | Glucose | G | A | 0.822 | 0.011 | 0.018 | -0.014 | 0.030 | -0.038 | 0.039 |
| rs6113722 | Glucose | G | A | 0.953 | -0.051 | 0.028 | -0.064 | 0.045 | -0.023 | 0.063 |
| rs6975024 | Glucose | T | C | 0.847 | 0.013 | 0.017 | -0.002 | 0.027 | -0.013 | 0.036 |
| rs749067 | Glucose | C | T | 0.351 | 0.016 | 0.014 | 0.009 | 0.022 | 0.009 | 0.028 |
| rs7633675 | Glucose | T | G | 0.673 | 0.003 | 0.013 | 0.022 | 0.022 | 0.023 | 0.030 |
| rs7756992 | Glucose | G | A | 0.287 | -0.014 | 0.014 | 0.011 | 0.022 | -0.001 | 0.030 |
| rs7903146 | Glucose | C | T | 0.728 | -0.001 | 0.014 | -0.002 | 0.022 | -0.020 | 0.029 |
| rs882020 | Glucose | C | T | 0.846 | -0.014 | 0.019 | -0.040 | 0.034 | 0.007 | 0.053 |
| rs983309 | Glucose | G | T | 0.873 | -0.006 | 0.019 | -0.031 | 0.036 | 0.033 | 0.059 |
| rs1019503 | 2h glucose | A | G | 0.496 | 0.003 | 0.013 | 0.003 | 0.021 | -0.012 | 0.027 |
| rs10934647 | 2h glucose | T | C | 0.235 | 0.014 | 0.016 | -0.014 | 0.029 | -0.022 | 0.045 |
| rs11672660 | 2h glucose | T | C | 0.218 | -0.010 | 0.016 | -0.005 | 0.026 | 0.028 | 0.035 |
| rs11782386 | 2h glucose | T | C | 0.124 | 0.021 | 0.021 | -0.002 | 0.035 | -0.053 | 0.046 |
| rs12255372 | 2h glucose | T | G | 0.263 | -0.021 | 0.014 | -0.031 | 0.022 | -0.013 | 0.030 |
| rs6547829 | 2h glucose | T | C | 0.095 | 0.030 | 0.022 | 0.003 | 0.036 | 0.005 | 0.048 |
| rs6975024 | 2h glucose | T | C | 0.847 | 0.013 | 0.017 | -0.002 | 0.027 | -0.013 | 0.036 |
| rs1005752 | T2D | A | C | 0.697 | 0.005 | 0.014 | 0.040 | 0.025 | -0.008 | 0.039 |
| rs10096633 | T2D | C | T | 0.878 | 0.000 | 0.017 | -0.022 | 0.028 | -0.016 | 0.038 |
| rs10097617 | T2D | T | C | 0.491 | 0.033 | 0.013 | 0.032 | 0.021 | 0.036 | 0.028 |
| rs10193538 | T2D | T | G | 0.590 | 0.002 | 0.013 | -0.026 | 0.024 | -0.011 | 0.036 |
| rs10195252 | T2D | T | C | 0.586 | -0.008 | 0.013 | 0.012 | 0.021 | -0.016 | 0.028 |
| rs10228066 | T2D | T | C | 0.524 | -0.016 | 0.013 | -0.005 | 0.021 | -0.004 | 0.028 |
| rs10406431 | T2D | A | G | 0.558 | 0.025 | 0.013 | 0.047 | 0.021 | 0.068 | 0.028 |
| rs1061810 | T2D | A | C | 0.289 | 0.025 | 0.014 | 0.033 | 0.022 | 0.022 | 0.030 |
| rs10750397 | T2D | A | G | 0.293 | 0.002 | 0.014 | -0.002 | 0.024 | 0.017 | 0.031 |
| rs10811660 | T2D | G | A | 0.830 | -0.004 | 0.017 | -0.031 | 0.027 | -0.038 | 0.036 |
| rs10830963 | T2D | G | C | 0.291 | 0.019 | 0.014 | 0.019 | 0.024 | 0.005 | 0.031 |
| rs10842994 | T2D | C | T | 0.808 | -0.024 | 0.017 | -0.062 | 0.027 | -0.052 | 0.035 |
| rs10882101 | T2D | T | C | 0.566 | -0.024 | 0.013 | -0.015 | 0.021 | -0.018 | 0.027 |
| rs10938398 | T2D | A | G | 0.426 | -0.002 | 0.013 | 0.025 | 0.021 | 0.016 | 0.027 |
| rs10954772 | T2D | T | C | 0.314 | 0.012 | 0.014 | 0.023 | 0.026 | 0.019 | 0.040 |
| rs10974438 | T2D | C | A | 0.357 | -0.005 | 0.013 | 0.007 | 0.022 | 0.032 | 0.028 |
| rs11063028 | T2D | C | T | 0.195 | -0.010 | 0.016 | -0.033 | 0.027 | 0.028 | 0.035 |
| rs11257655 | T2D | T | C | 0.231 | -0.040 | 0.017 | -0.074 | 0.027 | -0.076 | 0.034 |
| rs1127215 | T2D | C | T | 0.597 | 0.005 | 0.014 | 0.013 | 0.024 | 0.007 | 0.037 |
| rs11496066 | T2D | T | C | 0.819 | 0.016 | 0.017 | -0.009 | 0.026 | 0.006 | 0.035 |
| rs115505614 | T2D | T | C | 0.057 | 0.025 | 0.034 | 0.115 | 0.065 | 0.184 | 0.101 |
| rs11642430 | T2D | G | C | 0.395 | -0.019 | 0.013 | -0.039 | 0.022 | -0.025 | 0.029 |
| rs11680058 | T2D | A | G | 0.850 | 0.028 | 0.023 | 0.115 | 0.047 | 0.028 | 0.069 |
| rs11688682 | T2D | G | C | 0.735 | 0.009 | 0.018 | 0.046 | 0.033 | 0.005 | 0.046 |
| rs11699802 | T2D | C | T | 0.532 | -0.004 | 0.013 | -0.038 | 0.024 | -0.021 | 0.036 |
| rs11708067 | T2D | A | G | 0.787 | 0.015 | 0.016 | 0.049 | 0.025 | 0.047 | 0.034 |
| rs11709077 | T2D | G | A | 0.864 | -0.031 | 0.021 | -0.023 | 0.034 | -0.010 | 0.044 |
| rs11759026 | T2D | G | A | 0.239 | 0.005 | 0.017 | 0.001 | 0.030 | -0.007 | 0.042 |
| rs11842871 | T2D | G | T | 0.724 | -0.012 | 0.016 | -0.024 | 0.030 | -0.052 | 0.045 |
| rs12001437 | T2D | C | T | 0.363 | 0.024 | 0.013 | 0.047 | 0.021 | 0.071 | 0.028 |
| rs12140153 | T2D | G | T | 0.901 | 0.007 | 0.027 | 0.037 | 0.051 | 0.088 | 0.077 |
| rs1260326 | T2D | C | T | 0.630 | 0.016 | 0.013 | 0.033 | 0.021 | 0.030 | 0.028 |
| rs12640250 | T2D | C | A | 0.710 | -0.020 | 0.016 | 0.015 | 0.028 | -0.020 | 0.041 |
| rs12719778 | T2D | T | C | 0.549 | -0.015 | 0.013 | -0.035 | 0.021 | -0.040 | 0.028 |
| rs12811407 | T2D | A | G | 0.336 | 0.038 | 0.014 | 0.014 | 0.023 | 0.044 | 0.030 |
| rs12920022 | T2D | A | T | 0.172 | -0.030 | 0.020 | -0.022 | 0.033 | 0.062 | 0.050 |
| rs1296328 | T2D | A | C | 0.473 | -0.012 | 0.015 | -0.007 | 0.026 | 0.025 | 0.038 |
| rs13041756 | T2D | C | T | 0.111 | -0.013 | 0.020 | -0.031 | 0.033 | -0.022 | 0.044 |
| rs13262861 | T2D | C | A | 0.832 | 0.001 | 0.019 | -0.043 | 0.033 | -0.019 | 0.047 |
| rs13426680 | T2D | A | G | 0.924 | 0.011 | 0.023 | -0.014 | 0.038 | -0.054 | 0.056 |
| rs1359790 | T2D | G | A | 0.720 | -0.003 | 0.014 | 0.030 | 0.023 | 0.023 | 0.031 |
| rs1377807 | T2D | C | G | 0.306 | -0.024 | 0.015 | -0.006 | 0.027 | 0.044 | 0.039 |
| rs1412234 | T2D | C | T | 0.332 | 0.030 | 0.014 | 0.068 | 0.023 | 0.098 | 0.031 |
| rs141521721 | T2D | A | C | 0.027 | 0.057 | 0.047 | 0.103 | 0.072 | 0.087 | 0.091 |
| rs1421085 | T2D | C | T | 0.407 | 0.012 | 0.014 | 0.014 | 0.021 | -0.019 | 0.028 |
| rs1426371 | T2D | G | A | 0.720 | 0.003 | 0.017 | 0.002 | 0.030 | 0.002 | 0.043 |
| rs145678014 | T2D | G | T | 0.955 | -0.017 | 0.034 | -0.018 | 0.057 | 0.024 | 0.075 |
| rs145904381 | T2D | T | C | 0.979 | 0.008 | 0.077 | -0.008 | 0.156 | 0.273 | 0.227 |
| rs1493694 | T2D | T | C | 0.130 | 0.014 | 0.020 | -0.002 | 0.036 | 0.043 | 0.059 |
| rs1531583 | T2D | T | G | 0.059 | 0.021 | 0.026 | 0.093 | 0.044 | 0.022 | 0.065 |
| rs1561927 | T2D | C | T | 0.272 | -0.013 | 0.015 | 0.002 | 0.026 | -0.006 | 0.040 |
| rs1562396 | T2D | G | A | 0.314 | -0.014 | 0.016 | 0.021 | 0.028 | 0.019 | 0.040 |
| rs1580278 | T2D | C | A | 0.467 | -0.002 | 0.015 | 0.016 | 0.026 | -0.001 | 0.036 |
| rs1708302 | T2D | C | T | 0.530 | 0.033 | 0.013 | 0.035 | 0.021 | -0.011 | 0.028 |
| rs17122772 | T2D | G | C | 0.215 | -0.009 | 0.016 | 0.035 | 0.026 | 0.022 | 0.033 |
| rs17168486 | T2D | T | C | 0.196 | 0.020 | 0.017 | 0.012 | 0.028 | 0.027 | 0.036 |
| rs17250977 | T2D | G | A | 0.038 | -0.013 | 0.040 | 0.033 | 0.069 | -0.089 | 0.097 |
| rs17522122 | T2D | T | G | 0.469 | 0.018 | 0.013 | 0.003 | 0.024 | 0.049 | 0.036 |
| rs17684074 | T2D | G | C | 0.745 | -0.001 | 0.016 | -0.014 | 0.029 | -0.005 | 0.042 |
| rs17772814 | T2D | G | A | 0.904 | -0.012 | 0.030 | -0.019 | 0.055 | -0.129 | 0.084 |
| rs17791513 | T2D | A | G | 0.918 | -0.041 | 0.026 | 0.006 | 0.044 | -0.069 | 0.057 |
| rs1783541 | T2D | T | C | 0.176 | -0.011 | 0.016 | -0.006 | 0.026 | -0.008 | 0.034 |
| rs17836088 | T2D | C | G | 0.222 | 0.024 | 0.016 | 0.028 | 0.026 | 0.024 | 0.034 |
| rs1800961 | T2D | T | C | 0.043 | -0.057 | 0.038 | -0.059 | 0.062 | 0.015 | 0.083 |
| rs184509201 | T2D | C | G | 0.970 | 0.022 | 0.055 | 0.006 | 0.086 | 0.160 | 0.109 |
| rs2102278 | T2D | G | A | 0.339 | -0.012 | 0.016 | -0.010 | 0.025 | -0.037 | 0.030 |
| rs2197973 | T2D | T | C | 0.553 | -0.015 | 0.013 | -0.017 | 0.021 | -0.048 | 0.028 |
| rs2237895 | T2D | C | A | 0.438 | 0.002 | 0.013 | -0.008 | 0.021 | 0.033 | 0.028 |
| rs2258238 | T2D | T | A | 0.108 | 0.015 | 0.019 | 0.014 | 0.030 | -0.021 | 0.042 |
| rs2268078 | T2D | A | G | 0.670 | -0.019 | 0.015 | -0.016 | 0.026 | -0.012 | 0.040 |
| rs2272163 | T2D | C | A | 0.628 | 0.008 | 0.015 | 0.043 | 0.027 | 0.032 | 0.038 |
| rs2283220 | T2D | A | G | 0.670 | 0.014 | 0.014 | 0.024 | 0.026 | 0.030 | 0.040 |
| rs2307111 | T2D | T | C | 0.588 | -0.015 | 0.013 | -0.003 | 0.021 | 0.002 | 0.028 |
| rs243024 | T2D | A | G | 0.460 | -0.003 | 0.013 | -0.005 | 0.021 | -0.032 | 0.027 |
| rs2767036 | T2D | C | A | 0.305 | 0.012 | 0.014 | 0.029 | 0.025 | 0.012 | 0.038 |
| rs2796441 | T2D | G | A | 0.596 | 0.016 | 0.013 | 0.012 | 0.021 | 0.026 | 0.028 |
| rs28505901 | T2D | G | A | 0.748 | -0.007 | 0.016 | -0.016 | 0.028 | -0.033 | 0.043 |
| rs2972144 | T2D | G | A | 0.641 | 0.000 | 0.013 | -0.017 | 0.021 | -0.030 | 0.029 |
| rs3111316 | T2D | A | G | 0.600 | -0.004 | 0.013 | -0.017 | 0.021 | -0.021 | 0.028 |
| rs329122 | T2D | A | G | 0.431 | 0.010 | 0.013 | 0.000 | 0.024 | 0.002 | 0.037 |
| rs340874 | T2D | C | T | 0.525 | -0.010 | 0.014 | -0.028 | 0.024 | -0.013 | 0.035 |
| rs34584161 | T2D | A | G | 0.758 | 0.001 | 0.015 | -0.021 | 0.025 | 0.035 | 0.033 |
| rs34715063 | T2D | C | T | 0.120 | -0.006 | 0.022 | -0.020 | 0.041 | -0.091 | 0.061 |
| rs348330 | T2D | G | A | 0.372 | -0.003 | 0.015 | -0.003 | 0.027 | -0.014 | 0.039 |
| rs34965774 | T2D | A | G | 0.160 | 0.015 | 0.020 | 0.058 | 0.034 | 0.020 | 0.052 |
| rs35352848 | T2D | T | C | 0.766 | 0.005 | 0.015 | 0.014 | 0.025 | -0.001 | 0.034 |
| rs35895680 | T2D | C | A | 0.689 | -0.008 | 0.015 | -0.011 | 0.027 | -0.006 | 0.040 |
| rs35999103 | T2D | T | C | 0.169 | -0.010 | 0.017 | -0.026 | 0.029 | -0.027 | 0.039 |
| rs3751837 | T2D | T | C | 0.223 | -0.012 | 0.018 | -0.029 | 0.032 | 0.019 | 0.044 |
| rs3768321 | T2D | T | G | 0.189 | 0.008 | 0.018 | 0.054 | 0.028 | 0.050 | 0.035 |
| rs3798519 | T2D | C | A | 0.200 | -0.005 | 0.016 | -0.011 | 0.027 | -0.027 | 0.036 |
| rs3802177 | T2D | G | A | 0.674 | 0.009 | 0.014 | -0.035 | 0.023 | -0.035 | 0.030 |
| rs3845281 | T2D | G | A | 0.904 | 0.027 | 0.022 | 0.040 | 0.036 | 0.074 | 0.049 |
| rs3887925 | T2D | T | C | 0.512 | 0.000 | 0.014 | 0.030 | 0.024 | 0.018 | 0.037 |
| rs4238013 | T2D | C | T | 0.224 | -0.007 | 0.018 | 0.006 | 0.032 | 0.005 | 0.045 |
| rs429358 | T2D | T | C | 0.840 | -0.004 | 0.019 | -0.032 | 0.030 | -0.002 | 0.041 |
| rs4457053 | T2D | G | A | 0.281 | 0.008 | 0.014 | -0.009 | 0.022 | 0.039 | 0.030 |
| rs465002 | T2D | T | C | 0.716 | 0.005 | 0.015 | -0.018 | 0.024 | -0.022 | 0.031 |
| rs4688760 | T2D | T | C | 0.672 | 0.017 | 0.014 | 0.049 | 0.026 | 0.014 | 0.039 |
| rs4709746 | T2D | C | T | 0.875 | 0.003 | 0.020 | 0.030 | 0.036 | -0.031 | 0.053 |
| rs474513 | T2D | A | G | 0.519 | -0.001 | 0.013 | -0.011 | 0.021 | -0.024 | 0.027 |
| rs4804833 | T2D | A | G | 0.393 | 0.008 | 0.013 | -0.034 | 0.024 | -0.018 | 0.037 |
| rs4925109 | T2D | A | G | 0.336 | 0.002 | 0.015 | 0.040 | 0.027 | 0.066 | 0.037 |
| rs4929965 | T2D | A | G | 0.372 | -0.005 | 0.013 | 0.031 | 0.022 | 0.039 | 0.029 |
| rs4932265 | T2D | T | C | 0.265 | -0.023 | 0.016 | 0.008 | 0.028 | -0.008 | 0.042 |
| rs4946812 | T2D | G | A | 0.667 | -0.018 | 0.016 | -0.032 | 0.028 | -0.112 | 0.040 |
| rs4977213 | T2D | C | T | 0.349 | 0.032 | 0.014 | 0.038 | 0.026 | 0.058 | 0.039 |
| rs539515 | T2D | C | A | 0.197 | 0.016 | 0.016 | 0.061 | 0.026 | 0.036 | 0.036 |
| rs55653563 | T2D | A | C | 0.740 | -0.002 | 0.016 | -0.027 | 0.024 | -0.020 | 0.031 |
| rs56337234 | T2D | C | T | 0.485 | 0.002 | 0.013 | -0.015 | 0.024 | -0.024 | 0.038 |
| rs56348580 | T2D | G | C | 0.712 | 0.018 | 0.014 | 0.016 | 0.023 | 0.000 | 0.030 |
| rs58432198 | T2D | C | T | 0.890 | -0.001 | 0.022 | -0.007 | 0.039 | 0.080 | 0.058 |
| rs58730668 | T2D | T | C | 0.864 | 0.020 | 0.019 | 0.008 | 0.030 | 0.015 | 0.039 |
| rs601945 | T2D | G | A | 0.168 | -0.067 | 0.019 | -0.066 | 0.031 | -0.089 | 0.040 |
| rs60276348 | T2D | T | C | 0.151 | 0.008 | 0.019 | 0.028 | 0.034 | 0.060 | 0.049 |
| rs61676547 | T2D | C | G | 0.215 | -0.024 | 0.016 | -0.005 | 0.028 | 0.012 | 0.043 |
| rs62007683 | T2D | G | T | 0.672 | 0.015 | 0.015 | 0.062 | 0.026 | 0.014 | 0.038 |
| rs62080313 | T2D | C | T | 0.113 | -0.040 | 0.019 | -0.056 | 0.030 | -0.049 | 0.040 |
| rs62107261 | T2D | T | C | 0.953 | -0.033 | 0.032 | -0.056 | 0.050 | -0.025 | 0.065 |
| rs62271373 | T2D | A | T | 0.055 | -0.031 | 0.033 | -0.004 | 0.058 | 0.036 | 0.082 |
| rs6458354 | T2D | C | T | 0.298 | 0.013 | 0.014 | 0.041 | 0.025 | 0.060 | 0.040 |
| rs6459733 | T2D | G | C | 0.671 | 0.000 | 0.014 | 0.009 | 0.025 | 0.034 | 0.038 |
| rs6518681 | T2D | G | A | 0.905 | 0.027 | 0.025 | 0.050 | 0.046 | 0.043 | 0.072 |
| rs6600191 | T2D | T | C | 0.829 | -0.009 | 0.016 | 0.013 | 0.025 | -0.008 | 0.034 |
| rs67232546 | T2D | T | C | 0.196 | 0.017 | 0.017 | 0.071 | 0.029 | 0.003 | 0.044 |
| rs6780171 | T2D | A | T | 0.322 | -0.010 | 0.014 | -0.033 | 0.022 | -0.027 | 0.030 |
| rs6821438 | T2D | A | G | 0.539 | 0.005 | 0.014 | 0.016 | 0.025 | -0.020 | 0.036 |
| rs6976111 | T2D | A | C | 0.329 | 0.007 | 0.016 | 0.035 | 0.028 | 0.040 | 0.042 |
| rs7022807 | T2D | G | A | 0.398 | 0.000 | 0.013 | -0.014 | 0.021 | -0.024 | 0.028 |
| rs702634 | T2D | A | G | 0.685 | -0.010 | 0.014 | -0.049 | 0.023 | -0.076 | 0.030 |
| rs71372253 | T2D | C | T | 0.063 | 0.010 | 0.027 | -0.041 | 0.044 | -0.046 | 0.055 |
| rs7178762 | T2D | C | T | 0.493 | -0.007 | 0.013 | 0.000 | 0.022 | 0.004 | 0.027 |
| rs7222481 | T2D | C | G | 0.322 | -0.017 | 0.015 | -0.065 | 0.026 | -0.059 | 0.041 |
| rs7240767 | T2D | C | T | 0.388 | 0.014 | 0.014 | -0.008 | 0.023 | -0.016 | 0.029 |
| rs72802342 | T2D | C | A | 0.919 | -0.032 | 0.027 | -0.077 | 0.048 | -0.028 | 0.070 |
| rs72926932 | T2D | C | A | 0.089 | 0.014 | 0.025 | 0.043 | 0.042 | 0.017 | 0.054 |
| rs738408 | T2D | T | C | 0.228 | -0.036 | 0.016 | -0.077 | 0.029 | -0.113 | 0.044 |
| rs7629630 | T2D | A | T | 0.849 | -0.016 | 0.017 | -0.010 | 0.028 | -0.016 | 0.039 |
| rs7669833 | T2D | T | A | 0.691 | 0.001 | 0.015 | 0.023 | 0.027 | -0.038 | 0.039 |
| rs76895963 | T2D | T | G | 0.972 | -0.084 | 0.064 | -0.029 | 0.142 | -0.169 | 0.198 |
| rs7719891 | T2D | G | A | 0.267 | 0.038 | 0.016 | 0.002 | 0.028 | 0.013 | 0.040 |
| rs77464186 | T2D | A | C | 0.820 | -0.008 | 0.018 | -0.031 | 0.030 | -0.088 | 0.040 |
| rs7756992 | T2D | G | A | 0.287 | -0.014 | 0.014 | 0.011 | 0.022 | -0.001 | 0.030 |
| rs77864822 | T2D | A | G | 0.922 | 0.001 | 0.029 | -0.041 | 0.053 | 0.080 | 0.081 |
| rs7903146 | T2D | T | C | 0.272 | 0.001 | 0.014 | 0.002 | 0.022 | 0.020 | 0.029 |
| rs7987740 | T2D | T | C | 0.599 | 0.011 | 0.013 | 0.019 | 0.021 | 0.011 | 0.028 |
| rs8010382 | T2D | G | A | 0.431 | -0.013 | 0.015 | -0.022 | 0.025 | -0.036 | 0.037 |
| rs80147536 | T2D | A | T | 0.913 | -0.004 | 0.023 | 0.021 | 0.037 | 0.040 | 0.048 |
| rs8107974 | T2D | T | A | 0.082 | -0.008 | 0.023 | 0.010 | 0.038 | 0.022 | 0.052 |
| rs878521 | T2D | A | G | 0.239 | -0.017 | 0.016 | -0.009 | 0.025 | -0.006 | 0.031 |
| rs9379084 | T2D | G | A | 0.885 | 0.009 | 0.022 | -0.015 | 0.037 | -0.008 | 0.053 |
| rs9494624 | T2D | A | G | 0.291 | -0.009 | 0.015 | -0.006 | 0.027 | 0.013 | 0.042 |
| rs9563615 | T2D | A | T | 0.691 | 0.018 | 0.016 | 0.051 | 0.026 | 0.056 | 0.040 |
| rs9860730 | T2D | A | G | 0.677 | 0.012 | 0.014 | 0.002 | 0.025 | 0.001 | 0.038 |
| rs9873618 | T2D | G | A | 0.713 | 0.001 | 0.014 | 0.002 | 0.025 | 0.000 | 0.039 |
| rs9957145 | T2D | G | A | 0.807 | 0.027 | 0.016 | 0.009 | 0.027 | 0.035 | 0.036 |

EAF: Effect allele frequency; SE: Standard error; SNP: Single nucleotide polymorphism; T2D: Type 2 diabetes

**Additional file 1: Table S3: Studies related to COVID-19 analysis, as per extracted from COVID-19 Host Genetics Initiative (https://www.covid19hg.org/)**

| Study name | Setting | Case | Control | *Design | Case ascertainment |
| --- | --- | --- | --- | --- | --- |
| UK Biobank | Stockport, United Kingdom | 1439 | 385554 | Retrospective | Data linkage to Hospital Episode Statistics, intensive care unit data and mortality data |
| deCODE | Reykjavik, Iceland | 1897 | 273257 | Retrospective | Not described in details |
| Estonian Biobank | Tartu, Estonia | 313 | 138272 | Retrospective, Prospective | Linkage to electronic health records |
| GEN-COVID, reCOVID | Santiago de Compostela, Spain & Siena, Italy | 734 | 2472 | Retrospective, Prospective | Laboratory confirmed cases |
| FinnGen | Helsinki, Finland | 357 | 238354 | Retrospective | Based on infection registry |
| Genomics England | London, United Kingdom | 218 | 62302 | N/A | COVID-19 test results from Nati |
| Determining the Molecular Pathways and Genetic Predisposition of the Acute Inflammatory Process Caused by SARS-CoV-2 | Granada, Spain | 362 | 302 | Prospective | PCR verified cases at hospital |
| Genes & Health | London, United Kingdom | 114 | 27301 | Retrospective | Based on hospital records |
| Helix Exome+ COVID-19 Phenotypes | San Mateo, United States | 178 | 5441 | Retrospective | Online survey |
| Corea (Genetics of COVID-related Manifestation) | Seoul, Korea | 108 | 6500 | Retrospective | Not described in details although linkage to electronic medical records and laboratory test data was mentioned |
| Lifelines | Groningen, The Netherlands | 358 | 25213 | Retrospective | Online survey and hospital records |
| Michigan Genomics Initiative | Ann Arbor, United States | 122 | 51458 | Retrospective, Prospective | Self-identified or based on records in the Michigan health system |
| Million Veterans Program | Boston, United States | 3247 | 16235 | Retrospective | Not described in details but possibly based on electronic health records and questionnaires |
| Netherlands Twin Register | Amsterdam, The Netherlands | 145 | 5252 | Retrospective, Prospective | Record linkage on COVID-19 test |
| Partners Healthcare Biobank | Boston, United States | 277 | 34816 | Retrospective, Prospective | Not described in details |
| Penn Medicine Biobank | Philadelphia, United States | 166 | 8436 | Retrospective, Prospective | Based on electronic health records |
| Qatar Genome Program | Doha, Qatar | 700 | 13360 | Retrospective | Not described in details |
| Genomic epidemiology of SARS-Cov-2 and host genetics in Coronavirus Disease 2019 (COVID-19) | Stanford, United States | 109 | 191 | Retrospective, Prospective | Not described in details |
| UK Blood Donors Cohort | Cambridge, United Kingdom | 161 | 41674 | Retrospective | Linkage to health records |
| Biobanque Quebec COVID19 | Montreal, Canada | 206 | 327 | Prospective | Based on COVID-19 test |
| Ancestry | San Francisco, United States | 2417 | 14933 | Retrospective | Online survey |
| Amsterdam UMC COVID study group | Amsterdam, The Netherlands | 108 | 1413 | Prospective | Hospital confirmed cases |
| Genetic determinants of COVID-19 complications in the Brazilian population | Sao Paulo, Brazil | 756 | 1637 | Prospective | Based on SARS-CoV-2 RNA-based test |
| Genetic modifiers for COVID-19 related illness | Bruxelles, Belgium | 109 | 1484 | Prospective | Not described in details |
| COVID19-Host(a)ge | Kiel, Germany | 1610 | 2205 | Retrospective | Not described in detail. Samples from hotspots in Italy and Spain |
| The genetic predisposition to severe COVID-19 | Stockholm, Sweden | 78 | 3778 | Prospective | PCR verified cases at hospital |
| genomiCC | Kingston, Canada | 1676 | 8380 | Prospective | Hospital confirmed cases |
|  |  |  |  |  |  |
| All studies |  | 17,965 | 1,370,547 |  |  |

*For definition of design, please refer to https://www.covid19hg.org/about/

**Additional file 1: Table S4: Studies related to hospitalized COVID-19 analysis, as per extracted from COVID-19 Host Genetics Initiative (https://www.covid19hg.org/)**

| Study name | Setting | Case | Control | *Design | Case ascertainment |
| --- | --- | --- | --- | --- | --- |
| Amsterdam UMC COVID study group | Amsterdam, The Netherlands | 108 | 1413 | Prospective | Hospital confirmed cases |
| deCODE | Reykjavik, Iceland | 89 | 274322 | Retrospective | Not described in details |
| Genetic determinants of COVID-19 complications in the Brazilian population | Sao Paulo, Brazil | 756 | 1637 | Prospective | Based on SARS-CoV-2 RNA-based test |
| Genetic modifiers for COVID-19 related illness | Bruxelles, Belgium | 109 | 1484 | Prospective | Not described in details |
| GEN-COVID, reCOVID | Santiago de Compostela, Spain & Siena, Italy | 571 | 2472 | Retrospective, Prospective | Laboratory confirmed cases |
| FinnGen | Helsinki, Finland | 83 | 238628 | Retrospective | Based on infection registry |
| Determining the Molecular Pathways and Genetic Predisposition of the Acute Inflammatory Process Caused by SARS-CoV-2 | Granada, Spain | 311 | 302 | Prospective | PCR verified cases at hospital |
| Genes & Health | London, United Kingdom | 62 | 27353 | Retrospective | Based on hospital record |
| COVID19-Host(a)ge | Kiel, Germany | 1610 | 2205 | Retrospective | Not described in detail. Samples from hotspots in Italy and Spain |
| Corea (Genetics of COVID-related Manifestation) | Seoul, Korea | 69 | 6500 | Retrospective | Not described in details although linkage to electronic medical records and laboratory test data was mentioned |
| Penn Medicine Biobank | Philadelphia, United States | 66 | 8536 | Retrospective, Prospective | Based on electronic health records |
| Qatar Genome Program | Doha, Qatar | 60 | 13360 | Retrospective | Not described in details |
| Biobanque Quebec COVID19 | Montreal, Canada | 181 | 354 | Prospective | Based on COVID-19 test |
| UK Biobank | Stockport, United Kingdom | 765 | 364341 | Retrospective | Data linkage to Hospital Episode Statistics, intensive care unit data and mortality data |
| Million Veterans Program | Boston, United States | 902 | 4510 | Retrospective | Not described in details but possibly based on electronic health records and questionnaires |
| Bonn Study of COVID19 genetics | Bonn, Germany | 139 | 262 | Retrospective, Prospective | Not described in details |
| Ancestry | San Francisco, United States | 250 | 1967 | Retrospective | Online survey |
| The genetic predisposition to severe COVID-19 | Stockholm, Sweden | 78 | 3778 | Prospective | PCR verified hospital cases |
| genomiCC | Kingston, Canada | 1676 | 8380 | Prospective | Hospital confirmed cases |
|  |  |  |  |  |  |
| All studies |  | 7,885 | 961,804 |  |  |

*For definition of design, please refer to https://www.covid19hg.org/about/

**Additional file 1: Table S5: Studies related to severe COVID-19 analysis, as per extracted from COVID-19 Host Genetics Initiative (https://www.covid19hg.org/)**

| Study name | City and country | Case | Control | *Design | Case ascertainment |
| --- | --- | --- | --- | --- | --- |
| Amsterdam UMC COVID Study Group | Amsterdam, The Netherlands | 66 | 1413 | Prospective | Hospital confirmed cases |
| Genetic determinants of COVID-19 complications in the Brazilian population | Sao Paulo, Brazil | 450 | 1637 | Prospective | Based on SARS-CoV-2 RNA-based test |
| GEN-COVID, reCOVID | Santiago de Compostela, Spain & Siena, Italy | 468 | 2472 | Retrospective, Prospective | Laboratory confirmed cases |
| The genetic predisposition to severe COVID-19 | Stockholm, Sweden | 78 | 3778 | Prospective | PCR verified cases at hospital |
| FinnGen | Helsinki, Finland | 54 | 238657 | Retrospective | Based on infection registry |
| genomiCC | Kingston, Canada | 1676 | 8380 | Prospective | Hospital confirmed cases |
| Determining the Molecular Pathways and Genetic Predisposition of the Acute Inflammatory Process Caused by SARS-CoV-2 | Granada, Spain | 101 | 302 | Prospective | PCR verified cases at hospital |
| Biobanque Quebec COVID19 | Montreal, Canada | 55 | 480 | Prospective | Based on COVID-19 test |
| UK Biobank | Stockport, United Kingdom | 329 | 364341 | Retrospective | Data linkage to Hospital Episode Statistics. ICU data and mortality data |
| Italy COVID19-Host(a)ge | Kiel, Germany | 698 | 1255 | Retrospective | Not described in detail. Samples from hotspots in Italy and Spain |
| Spain COVID19-Host(a)ge | Kiel, Germany | 302 | 925 | Retrospective | Not described in detail. Samples from hotspots in Italy and Spain |
| Bonn Study of COVID19 genetics | Bonn, Germany | 59 | 262 | Retrospective, Prospective | Not described in detail. |
|  |  |  |  |  |  |
| All studies |  | 4,336 | 623,902 |  |  |

*For definition of design, please refer to https://www.covid19hg.org/about/

**Additional file 1: Table S6: Definition of the COVID-19 phenotypes, as per extracted from COVID-19 Host Genetics Initiative (https://www.covid19hg.org/)**

| **Phenotype** | **Case ascertainment** |
| --- | --- |
| COVID-19 | Individuals with laboratory confirmation of SARS-CoV-2 infection (RNA and/or serology based) OR EHR/ICD coding/ Physician Confirmed COVID-19 OR self-reported COVID-19 positive (e.g. by questionnaire) |
| Hospitalized COVID-19 | Hospitalized laboratory confirmed SARS-CoV-2 infection (RNA and/or serology based), hospitalization due to corona-related symptoms. |
| Severe COVID-19 | Hospitalized laboratory confirmed SARS-CoV-2 infection (RNA and/or serology based), AND (death OR respiratory support (intubation, CPAP, BiPAP, CNP (continue external negative pressure), Optiflow/very high flow Positive End Expiratory Pressure Oxygen* - AND hospitalization with COVID19 as primary reason for admission.  *Simple supplementary oxygen (e.g. 2 litres/minute via nasal cannulae) only does not qualify for case status. |

**Additional file 1: Table S7: Minimum detectable odds ratio per standard deviation of the exposure in this Mendelian randomization, at 80% power and 5% statistical significance**

|  | Variance explained by all instruments | Any COVID-19 | Hospitalized COVID-19 | Severe COVID-19 |
| --- | --- | --- | --- | --- |
| Exposure |  |  |  |  |
| HbA1c | 2.2% | 1.15 | 1.25 | 1.35 |
| Glucose | 2.6% | 1.14 | 1.23 | 1.30 |
| 2 hour glucose | 0.7% | 1.30 | 1.50 | 1.70 |
| Liability to type 2 diabetes | 4.4% | 1.11 | 1.17 | 1.23 |

**Additional file 1: Figure S1: Association of genetically predicted glycemic traits and genetic predisposition to type 2 diabetes on risk of COVID-19 using Mendelian randomization, excluding instruments related to body mass index**

**

**Additional file 1: Figure S2: Association of genetically predicted glycemic traits and genetic predisposition to type 2 diabetes on risk of hospitalized COVID-19 using Mendelian randomization excluding instruments related to body mass index**

**

**Additional file 1: Figure S3: Association of genetically predicted glycemic traits and genetic predisposition to type 2 diabetes on risk of severe COVID-19 using Mendelian randomization excluding instruments related to body mass index**

**
